# In Memoriam—John C. Robert, M. D.

**Published:** 1894-01

**Authors:** J. W. Thompson

**Affiliations:** Secretary pro tem.


### IN MEMORIAM—DR. JOHN C. ROBERT.

At the regular monthly meeting of the New York Homœopathic Union, held at 53 West Forty-fifth Street, New York, November 16th, 1893, the President, Edmund Carleton, M. D., in the chair, announcement was made of the death of John C. Robert, M. D., at New Utrecht, N. Y., on the 12th inst.

After remarks by members upon the character and services of the deceased, the following resolutions were offered by B. Fincke, M. D., seconded, and by vote unanimously adopted:

WHEREAS, It has pleased God to remove from us Dr. John C. Robert, of New Utrecht, a graduate of Bellevue Hospital College, member of the New York Homœopathic Union and of the International Hahnemannian Association;

*Resolved*, That by his death we have lost a true Hahnemannian homœopathician, who in his quiet, unassuming way contributed to the promotion of homœopathic science and art, by careful provings and successful practice;

*Resolved*, That we deem a public recognition due to his memory, in this behalf, and also on account of his philanthropic work among the sick and suffering lowly;

*Resolved*, That these resolutions be entered upon the minutes of the Union, and that copies be sent to his family and to the homœopathic journals.

(Signed) J. W. THOMPSON, M. D.,
*Secretary pro tem.*

